# Critical Role of Activating Transcription Factor 4 in the Anabolic Actions of Parathyroid Hormone in Bone

**DOI:** 10.1371/journal.pone.0007583

**Published:** 2009-10-23

**Authors:** Shibing Yu, Renny T. Franceschi, Min Luo, Jie Fan, Di Jiang, Huiling Cao, Tae-Geon Kwon, Yumei Lai, Jian Zhang, Kenneth Patrene, Kurt Hankenson, G. David Roodman, Guozhi Xiao

**Affiliations:** 1 Department of Medicine, University of Pittsburgh, Pittsburgh, Pennsylvania, United States of America; 2 Department of Surgery, University of Pittsburgh, Pittsburgh, Pennsylvania, United States of America; 3 Department of Pharmacology and Chemical Biology, University of Pittsburgh, Pittsburgh, Pennsylvania, United States of America; 4 Department of Periodontics and Oral Medicine, University of Michigan, Ann Arbor, Michigan, United States of America; 5 Department of Biological Chemistry, School of Dentistry, University of Michigan, Ann Arbor, Michigan, United States of America; 6 Department of Medicine, School of Medicine, University of Michigan, Ann Arbor, Michigan, United States of America; 7 Department of Oral and Maxillofacial Surgery, School of Dentistry, Kyungpook National University, Daegu, Korea; 8 Department of Animal Biology, School of Veterinary Medicine, University of Pennsylvania, Philadelphia, Pennsylvania, United States of America; Pennsylvania State University, United States of America

## Abstract

Parathyroid hormone (PTH) is a potent anabolic agent for the treatment of osteoporosis. However, its mechanism of action in osteoblast and bone is not well understood. In this study, we show that the anabolic actions of PTH in bone are severely impaired in both growing and adult ovariectomized mice lacking bone-related activating transcription factor 4 (ATF4). Our study demonstrates that ATF4 deficiency suppresses PTH-stimulated osteoblast proliferation and survival and abolishes PTH-induced osteoblast differentiation, which, together, compromise the anabolic response. We further demonstrate that the PTH-dependent increase in osteoblast differentiation is correlated with ATF4-dependent up-regulation of Osterix. This regulation involves interactions of ATF4 with a specific enhancer sequence in the Osterix promoter. Furthermore, actions of PTH on Osterix require this same element and are associated with increased binding of ATF4 to chromatin. Taken together these experiments establish a fundamental role for ATF4 in the anabolic actions of PTH on the skeleton.

## Introduction

Parathyroid hormone (PTH) is a major regulator of calcium homeostasis and has both catabolic and anabolic effects on osteoblasts and bone that depend on the temporal pattern of administration. Continuous administration of PTH decreases bone mass whereas intermittent administration increases bone mass [1]–[6]. The mechanism(s) responsible for these differing effects are poorly understood. The anabolic activity of PTH has been attributed to both direct actions of this hormone on osteoprogenitor cells as well as indirect effects mediated by the production of growth factors such as insulin-like growth factor-1 (IGF-1) and basic fibroblast growth factor-2 (FGF-2) [7], [8]. Most cellular actions of PTH are mediated by the PTH-1 receptor, a G protein-coupled receptor that is expressed in osteoblasts [9], [10]. Binding of PTH to its receptor activates multiple intracellular signaling pathways that involve cAMP, inositol phosphates, intracellular Ca^2+^, protein kinases A and C [11], and the extracellular signal-related (ERK)/mitogen-activated protein kinase (MAPK) pathway [12], [13]. Activation of these signal transduction pathways ultimately affects cellular behavior. In this regard, the anabolic actions of PTH on bone have been attributed to increased proliferation of osteopregenitors/osteoblasts [2], [14], [15] and/or decreased osteoblast apoptosis [6], [16], [17].

Although a number of transcription factors including cAMP response element binding protein (CREB) [18], [19], AP1 family members [20]–[22], and Runx2 [20], [23] have been implicated in the molecular actions of PTH in osteoblasts, genetic studies have not strongly linked any of these factors in the anabolic actions of this hormone. To better understand the anabolic actions of PTH, it is essential that the downstream signals induced by this hormone be identified and evaluated for possible roles in bone formation. The *osteocalcin* (*Ocn*) promoter has been an important tool for unraveling the mechanisms mediating osteoblast-specific gene expression and was used to identify a number of important transcription factors and cofactors involved in *Ocn* gene expression [24]–[28]. Because the *Ocn* gene is regulated by PTH [29], [30], we have used it as a model system for identifying new transcriptional mediators of PTH action. We previously demonstrated that the OSE1 (osteoblast-specific element 1) in the proximal *mOG2* promoter [24] is necessary and sufficient for PTH induction of this gene [31]. The OSE1 core sequence (TTACATCA) was subsequently identified as a DNA binding site for the ATF4 transcription factor. The critical role of ATF4 in osteoblast differentiation and bone development was established using *Atf4*-deficient mice [26]. At the cellular level, ATF4 is critical for proliferation and differentiation as well as survival in osteoblasts [32], [33]. We recently showed that ATF4 is also required for PTH induction of *Ocn* expression in osteoblasts [34]. Specifically, PTH elevated levels of ATF4 mRNA and protein in a dose and time-dependent manner and increased binding of ATF4 to OSE1 DNA. Furthermore, PTH stimulation of *Ocn* expression was lost by siRNA downregulation of ATF4 in MC-4 cells and in primary bone marrow stromal cells from *Atf4^−/−^* mice. Collectively, these studies demonstrate that ATF4 is a novel downstream mediator of PTH signaling.

Osterix (Osx, or Sp7), a zinc-finger-containing transcription factor of the sp family, is essential for osteoblast differentiation and bone formation [35]. Since Osx is not detected in mice lacking Runx2 [35], a master regulator of osteoblast differentiation [25], [36]–[38], it functions downstream of Runx2. However, the molecular mechanisms whereby the Osx gene is transcriptionally regulated are not well understood.

In the present study, we used ATF4-deficient mice to determine whether ATF4 is more generally required for the in vivo anabolic actions of PTH in bone as well as explore the mechanism used by PTH to regulate ATF4 activity. As will be shown, loss of ATF4 greatly attenuated the anabolic effects of PTH. Furthermore, ATF4 may participate in the PTH response by regulating the expression of the Osterix transcription factor.

## Results

### The anabolic effects of PTH on bone are severely impaired in growing Atf4-deficient mice

**Figure 1:**
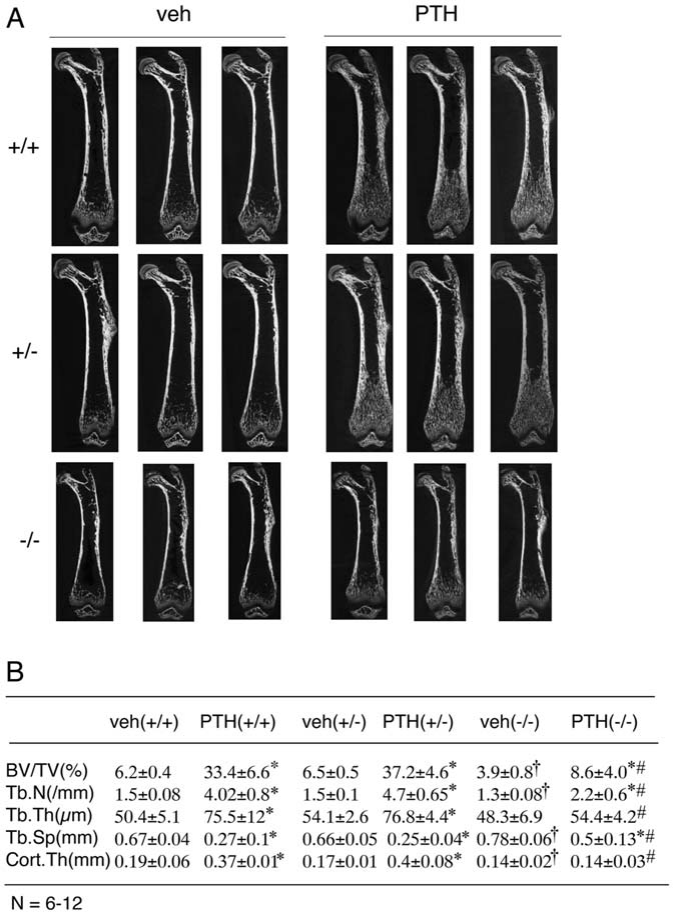
PTH-stimulated bone was significantly reduced or lost in Atf4^−/−^ femurs. A, two-dimensional (2D) reconstruction from μCT scan of femurs from growing *wt*, *Atf4^+/−^* and *Atf4^−/−^* mice treated with and without intermittent PTH for 28 d. B, quantitative analysis of bone volume/tissue volume (BV/TV), trabecular number (Tb. N), trabecular thickness (Tb.Th), trabecular space (Tb.Sp), and cortical thickness (Cort. Th). *P<0.05 (veh vs. PTH),^ †^ P<0.05 (wt-veh vs. *Atf4^−/−^*-veh), ^#^P<0.05 (PTH/veh-wt vs. PTH/veh-*Atf4^−/−^*).

We first evaluated our hypothesis that ATF4 mediates the anabolic actions of PTH in bone using a relatively simple “growing mouse” model system, that has been widely used for studying the anabolic actions of PTH, PTHrP, FGF2, and IGF-1 in bone [2]–[4], [8], [39], [40]. The advantages of this system are that it is less time consuming and costly versus adult ovarectomized mouse models. Furthermore, because young growing animals have relatively high osteoblast activity, they are more sensitive to PTH than adults [2], [11], [14], [16]–[18], [41]–[43]. Mice were treated with vehicle or PTH and sacrificed 24 h after the last PTH injection. PTH-dependent anabolic activity was evaluated in these mice using standard biochemical and histomorphometric criteria. *Atf4^−/−^* mice grew more slowly than wild type (wt) animals. The growth rate was slightly but significantly increased by PTH treatment during d 6–18 in wt but not *Atf4^−/−^* animals (Fig. S1A). *Atf4^−/−^* femurs were also shorter than *wt* or *Atf4^+/−^* femurs. Consistent with results from a previous study [3], PTH did not alter the length of femurs (Fig. S1B). However, it did significantly increase the dry ash weight per femur in wt and *Atf4^+/−^* but not in *Atf4^−/−^* mice (Fig. S1C). Serum Pi and calcium concentration (Fig. S1D and E) were not markedly affected by PTH or ATF4 deficiency. Faxitron X-ray analysis of femurs revealed that *wt* and *Atf4^+/−^* mice responded to PTH with markedly increased radiopacity throughout the whole femur, with the most dramatic increase in the metaphyseal region (Fig. S2, top and middle). In contrast, PTH only slightly increased the radiopacity in the same region of *Atf4^−/−^* femurs (Fig. S2, bottom). As shown in Fig. 1, quantitative μCT analysis of femur histomorphometric parameters showed that *Atf4^−/−^* mice had a significant reduction in bone volume/tissue volume (BV/TV), trabecular number (Tb.N), and cortical thickness (Cort.Th) and a marked increase in trabecular space (Tb.Sp) compared with the *wt* or *Atf4^+/−^* littermates. These data confirmed an essential role of ATF4 in bone that was previously demonstrated by the Karsenty group [26]. As expected, in *wt* femurs, intermittent PTH increased BV/TV, Tb.N, and Tb.Th by 5.4-fold, 2.7-fold, and 1.5-fold, respectively, and decreased Tb.Sp by 60 percent. Similar effects were also seen in *Atf4^+/−^* mice (Fig. 1B). In contrast, the PTH response was greatly attenuated in *Atf4^−/−^* mice where the following PTH responses were observed; BV/TV, 2.2-fold increase; Tb.N, 1.7-fold increase; Tb.Th, 1.1-fold increase; Tb.Sp, 36 percent decrease. In all cases, the magnitude of PTH-stimulated changes on BV/TV, Tb.N, Tb.Sp was dramatically reduced in *Atf4^−/−^* mice relative to *wt* or *Atf4^+/−^* mice (P<0.05, PTH/veh-wt vs. PTH/veh-*Atf4^−/−^*). Furthermore, PTH-stimulated increases in Cort.Th and Tb.Th were completely lost in *Atf4^−/−^* femurs. Because PTH similarly affected all trabecular and cortical parameters in *Atf4^+/+^* and *Atf4^+/−^* mice, subsequent experiments compared the PTH effects on bone only between *wt* and *Atf4^−/−^* mice.

**Figure 2 pone-0007583-g002:**
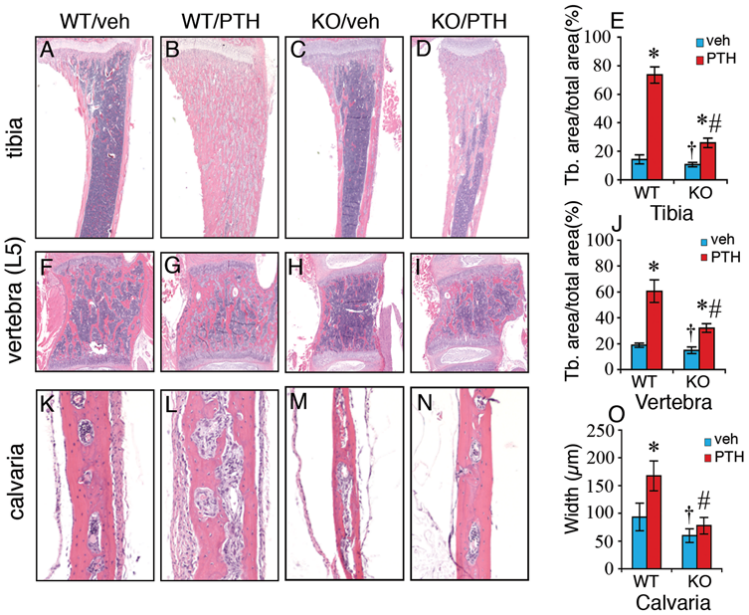
PTH-stimulated bone is severely impaired in Atf4^−/−^ tibiae, vertebrae, and calvariae. Representative H&E stained sections of tibiae (A–E), vertebrae (L5) (F–J), and calvariae (K–O) are shown. Trabecular bone area versus total area of tibiae (E) and vertebrae (J) was measured using an Image Pro Plus 6.2 software. The calvarial width was obtained from 20 random measurements throughout the whole calvaria using a SPOT Advanced imaging software (O). *P<0.05 (veh vs. PTH),^ †^ P<0.05 (wt-veh vs. *Atf4^−/−^*-veh), ^#^P<0.05 (PTH/veh-wt vs. PTH/veh-*Atf4^−/−^*).

We next measured effects of *Atf4* gene ablation on PTH stimulation of tibiae, vertebrae, and calvariae. The anabolic effect of PTH on wt tibiae was so dramatic that the majority of the bone marrow cavity was replaced by newly formed bone (Fig. 2A and B). In *Atf4* ^−/−^ tibiae, while PTH still induced a small increase in trabecular area, the magnitude of stimulation was significantly reduced (5-fold in wt vs. 2.2-fold in *Atf4^−/−^*)(P<0.05, PTH/veh-wt vs. PTH/veh-*Atf4^−/−^*)(Fig. 2C-E). Likewise, the PTH-stimulated increase in the trabeculae of vertebrae (L5) was markedly reduced in *Atf4* ^−/−^ mice (3-fold in wt vs. 2-fold in *Atf4^−/−^*) (P<0.05, PTH/veh-wt vs. PTH/veh-*Atf4^−/−^*)(Fig. 2F–J). When histological sections of calvariae were compared, PTH increased the width of the calvariae by 1.8-fold in *wt* mice, a response that was abolished in *Atf4* ^−/−^ animals (Fig. 2K–O).

### Ablation of the Atf4 gene impairs PTH stimulation of trabecular, but not cortical bone in 7-month-old ovariectomized (OVX) mice

**Figure 3:**
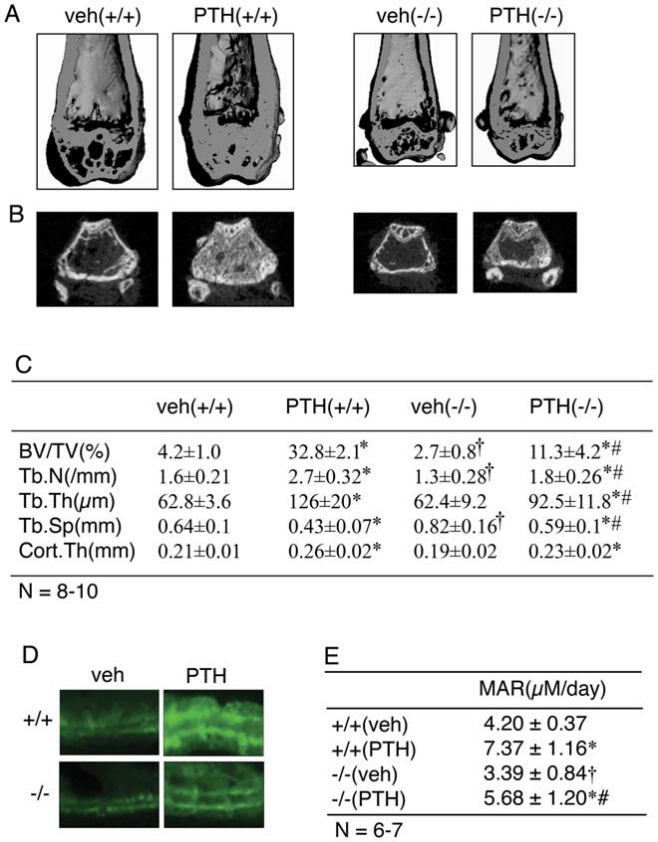
Effects of ATF4 deficiency on PTH stimulation in adult OVX bone. A, three-dimensional (3D) reconstruction from μCT scan of distal femurs of adult OVX mice. B, sagittal view of 2D distal femur at 1.7–2.0 mm from the chondro-osseous junction. C, BV/TV, Tb. N, Tb.Th, Tb.Sp, and Cort.Th. D, calcein double labeling of metaphyseal trabecular bone (magnification,x200). *P<0.05 (veh vs. PTH),^ †^ P<0.05 (wt-veh vs. *Atf4^−/−^*-veh), ^#^P<0.05 (PTH/veh-wt vs. PTH/veh-*Atf4^−/−^*).

The experiments described above clearly establish a critical role of ATF4 in the anabolic effects of PTH on long bones, vertebrae, and calvariae in rapidly growing mice. However, it is possible that results obtained from growing animals may be different from those in adults due to possible effects of PTH and/or ATF4 on animal growth or influences of animal growth on the anabolic response to PTH, either of which could complicate the interpretation of the results. In contrast, adult mice have a mature skeleton in which these possible complications can be avoided. Furthermore, the OVX mouse provides a model that may be more relevant to the clinical applications of PTH in the treatment of osteoporosis. For these reasons, we next evaluated whether ATF4 is required for the anabolic response to PTH in 7-month-old OVX mice. OVX surgery was successful as demonstrated by significant reduction in BV/TV (65 percent), Tb.N (27 percent), and Tb.Th (18 percent) and increased Tb.Sp (37 percent) relative to sham surgery (P<0.05, wt-sham vs. wt-OVX) (Fig. S3). OVX surgery did not reduce Cort.Th, which is consistent with results from rats [44], Interestingly, OVX surgery did not significantly reduce bone parameters in *Atf4^−/−^* animals (P>0.05, *Atf4^−/−^* -sham vs. *Atf4^−/−^* -OVX). As shown in Fig. 3 A–C, similar to results from growing mice, ablation of the *Atf4* gene significantly decreased BV/TV and Tb.N and increased Tb.Sp in adult OVX mice. In further agreement with results in young mice, *Atf4^−/−^* animals exhibited a clearly attenuated response to PTH. For example, while PTH increased BV/TV by 7.8-fold in wt mice, this value was only increased 4.2-fold in *Atf4^−/−^* animals. Similarly, while PTH still stimulated formation of trabecular bone in *Atf4^−/−^*trabeculae, the magnitude of this response was significantly reduced compared to wt control (P<0.05, PTH/veh-wt vs. PTH/veh- *Atf4^−/−^*). In contrast to result from growing mice, Cort.Th was not reduced by ablation of the *Atf4* gene in adult OVX mice (0.21±0.01 mm in wt vs. 0.19±0.02 mm in *Atf4^−/−^*, P>0.05 wt vs. *Atf4^−/−^*). Also, PTH was much less effective in stimulating Cort.Th in adult OVX mice (24%) than in growing mice (95%) (Figs. 1 and 3). Furthermore, no difference in stimulation of cortical thickness by PTH was observed when wt and *Atf4^−/−^* groups were compared (24% wt vs. 21% *Atf4^−/−^*)(P>0.05, PTH/veh-wt vs. PTH/veh-*Atf4^−/−^*). As shown in Fig. 3D and E, results from calcein double labeling of 7-month old OVX wt and *Atf4^−/−^* tibia revealed that the PTH-stimulated increase in mineral apposition rate (MAR), an indicator of osteoblast function, was significantly reduced by ATF4 deficiency (P<0.05, PTH/veh-wt vs. PTH/veh-*Atf4^−/−^*).

### ATF4 deficiency significantly reduces basal and PTH-stimulated proliferation in osteoblasts/preosteoblasts

**Figure 4:**
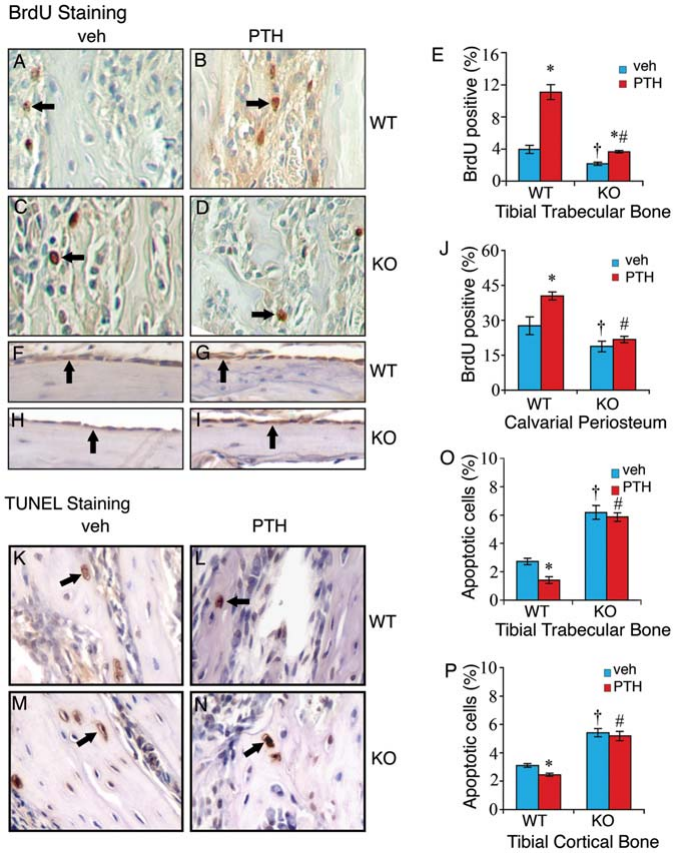
Effects of PTH on osteoblast proliferation and survival in wt and Atf4^−/−^ bone. A–J, BrdU staining, sections of tibiae (A–D) and calvariae (F–I) were stained using a Zymed BrdU immunostaining kit. Proliferating cells were stained brown (arrows) and non-proliferating cells were stained blue. Proliferating cells on tibial trabecular surface or osteoid (E) or calvarial periosteal surface (J) were counted and normalized to total cells from the same area. K–P, TUNEL staining, sections of tibiae were stained using the ApopTag Peroxidase *In Situ* Apoptosis Detection Kit. Apoptotic osteoblasts and osteocytes (arrows) were stained brown and non-apoptotic cells were stained blue. Apoptotic osteoblasts and osteocytes in the trabecular (O) and cortical bone (P) of tibiae were counted and normalized to total osteoblasts and osteocytes from the same area. *P<0.05 (veh vs. PTH),^ †^ P<0.05 (wt-veh vs. *Atf4^−/−^*-veh), ^#^P<0.05 (PTH/veh-wt vs. PTH/veh-*Atf4^−/−^*).

PTH and PTHrP are both known to increase the proliferation and numbers of osteoblasts [14], [15], [17], [45]. ATF4 is also a positive regulator of osteoblast proliferation and can be up-regulated by PTH in these cells [33], [34]. To determine whether ATF4 plays a role in PTH regulation of osteoblast proliferation, sections of tibiae and calvariae from wt and *Atf4* ^−/−^ mice treated with and without intermittent PTH were analyzed for in vivo cell proliferation using a Zymed BrdU immunostaining kit. As shown in Fig. 4A, B, and E, in wt mice, PTH increased the percentage of proliferating osteoblasts/preosteoblasts of tibial trabeculae by 2.8-fold relative to vehicle-treated control. Ablation of the *Atf4* gene resulted in a 50% decline in basal proliferation. In addition, the PTH-stimulated increase in proliferation was decreased by 40 percent (Fig. 4C–E). Similarly, PTH-induced proliferation in calvarial periosteal osteoblasts was also significantly reduced by ATF4 deficiency (Fig. 4F–J). As expected, very few osteocytes were BrdU-positive in both tibiae and calvariae. Note: basal proliferation rate of calvarial periosteal osteoblasts was significantly higher than that of tibial trabecular osteoblasts (28% vs. 4%). Therefore, ATF4 is critical for basal and PTH-stimulated proliferation of osteoblasts/preosteoblasts in vivo.

### PTH fails to reduce apoptotic death in Atf4^−/−^ osteoblasts/osteocytes

Mature osteoblasts synthesize and deposit a mineralizing extracellular matrix and become osteocytes. Both osteoblasts and osteocytes can be lost through apoptosis. PTH signaling increases the survival of osteoblasts and osteocytes by reducing apoptosis [6], [16], [17], [45]. Our recent study shows that ATF4 is anti-apoptotic in osteoblasts [33]. To determine whether ATF4 plays a role in PTH-mediated anti-apoptosis, sections of tibiae were stained with TUNEL and apoptotic cells were assessed. As shown in Fig. 4K and L, ATF4 deficiency significantly increased the basal levels of apoptosis. As expected, PTH dramatically reduced apoptotic death of tibial trabecular osteoblasts/osteocytes by 48 percent, which is consistent with results from previous studies [6], [16], [17], [45]. Importantly, the PTH-stimulated decrease in apoptotic death was completely abolished in *Atf4^−/−^* trabeculae (Fig. 4M–O). ATF4 was similarly required for PTH to inhibit apoptosis in cortical osteocytes of tibiae (Fig. 4P). Collectively, ATF4 is essential for PTH-mediated inhibition of apoptosis in osteoblasts/osteocytes in vivo.

### PTH-induced increase in expression of osteoblast differentiation marker genes is dramatically reduced or completely abolished in Atf4^−/−^ animals

**Figure 5:**
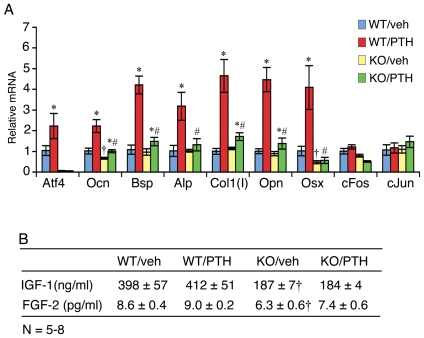
Effects of PTH on expression of osteoblast marker genes in wt and ATF4 deficient mice. A, quantitative real-time PCR, total RNAs were isolated from tibiae and analyzed by quantitative real-time RT-PCR using specific primers for *Atf4*, *Ocn*, *Bsp*, *Col 1(I)*, *ALP*, *Opn*, *Pthrp*, *c-Fos*, *and c-Jun* mRNAs, which were normalized to *Gapdh* mRNA. *P<0.05 (veh vs. PTH), ^#^P<0.05 (wt-veh vs. *Atf4^−/−^*-veh). B, plasma levels of IGF-1 and FGF-2 from mice using respective ELISA kits according to the manufacturer's instructions. *P<0.05 (veh vs. PTH),^†^P<0.05 (wt-veh vs. *Atf4^−/−^*-veh), ^#^P<0.05 (PTH/veh-wt vs. PTH/veh-*Atf4^−/−^*).

We next determined the effects of ATF4 deficiency on PTH induction of osteoblast differentiation markers in vivo. Total RNA was isolated from tibiae of *wt* and *Atf4* ^−/−^ mice treated with and without PTH for 28 d and expression levels of osteoblast differentiation marker genes were measured by quantitative real-time PCR analysis. As shown in Fig. 5A, PTH dramatically elevated the expression of genes known to be associated with osteoblast differentiation including *osteocalcin* (*Ocn*) (2.2-fold), *bone sialoprotein* (*Bsp*) (4.2-fold), *alkaline phosphatase* (*Alp*) (3.2-fold), *α1(I) collagen* (*Col1(I)*)(4.7-fold), *osteopontin* (*Opn*) (4.6-fold), and *osterix* (*Osx*) (4.1-fold). Importantly, this PTH regulation was either dramatically reduced or completely abolished in *Atf4^−/−^* tibiae. ATF4 deficiency also reduced basal *Ocn* and *Osx* mRNA levels. Consistent with our previous report [34], PTH increased *Atf4* mRNA 2.2-fold in wt tibiae, while *Atf4* was undetectable in *Atf4^−/−^* animals. In contrast, c-Fos and c-Jun, both early PTH-induced genes, were not induced by PTH in either wt or *Atf4^−/−^* tibiae. As shown in Fig. 5B, the levels of IGF-1 and FGF-2 which have both been implicated in the anabolic actions of PTH in bone [7], [8] were markedly reduced in plasma from *Atf4^−/−^* mice compared to wt mice (P<0.05, wt vs. *Atf4^−/−^*). However, their levels were not significantly elevated by the treatment of intermittent PTH in both wt or *Atf4^−/−^* animals (P>0.05, veh vs. PTH).

### Intermittent PTH increases in vivo Osx expression in osteoblasts through a pathway requiring ATF4

**Figure 6:**
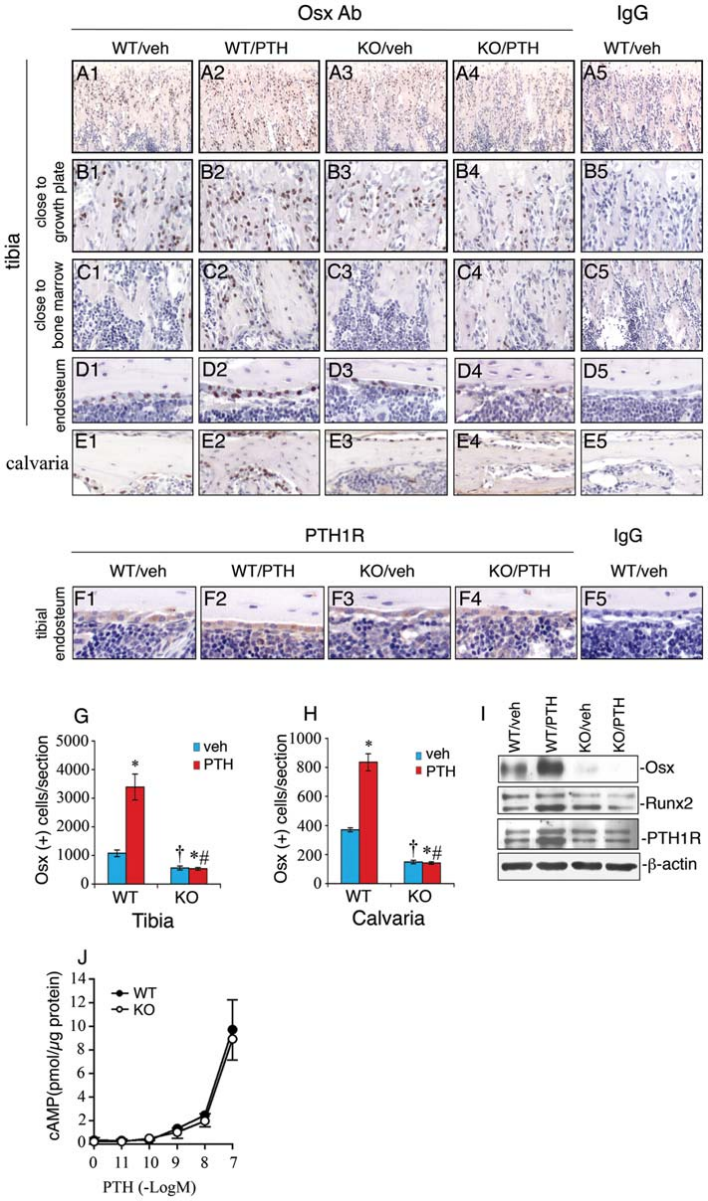
PTH fails to promote osteoblast maturation/differentiation in the absence of ATF4. A–E, IHC analysis of Osx expression, sections of tibiae (A–D) and calvariae (E) were immunohistochemically stained using a specific antibody against Osx protein. The nuclei of Osx-positive cells (i.e., osteoblasts) were stained brown. The nuclei of preosteoblasts and other cells are stained blue. The total numbers of Osx-positive osteoblasts per tibial (G) or calvarial (H) section were counted under microscope. F, sections of tibiae were stained using an antibody against PTH1R protein. I, Western blot analysis, protein extracts were isolated from tibiae and analyzed for Osx, Runx2, and PTH1R proteins. *P<0.05 (veh vs. PTH),^ †^ P<0.05 (wt-veh vs. *Atf4^−/−^*-veh), ^#^P<0.05 (PTH/veh-wt vs. PTH/veh-*Atf4^−/−^*). J, cAMP assay, primary calvarial osteoblasts from 3-d-old wt or *Atf4^−/−^* mice were isolated, seeded at density of 5×10^4^ on 96-well plate, and treated with vehicle or increasing concentrations of human recombinant PTH(1-34) for 5 min followed by measurement of cAMP.

Our above results demonstrate that ATF4 is essential for the major anabolic actions of PTH on bone and is also required for PTH-dependent induction of osteoblast differentiation. To begin to address the mechanism underlying this response, we measured the expression of Osterix (Osx) and Runx2 proteins, two critical transcription factors that regulate osteoblast differentiation. Initially, we used immunohistochemistry (IHC) to measure Osx in the tibiae and calvariae of wt and *Atf4^−/−^* mice with or without 28 d anabolic PTH treatment. As shown in Fig. 6, in wt-vehicle-treated tibiae, Osx-positive osteoblasts were only identified in the trabeculae and cortical endosteum close to the growth plate (Fig. 6A1, B1, and D1) and were almost undetectable in the same regions close to the marrow (Fig. 6C1), indicating that cells in these areas are still in the immature (preosteoblast) state. In contrast, in the wt-PTH group, Osx-positive osteoblasts were identified on all surfaces of trabeculae and endosteum throughout the tibia. PTH increased the total number of Osx-positive cells per tibial section by 3.2-fold in wt mice (Panel 2). ATF4 deficiency reduced the numbers of Osx-positive cells by 50 percent (Panel 3). Strikingly, although PTH slightly increased bone volume in *Atf4^−/−^* bone (Fig. 1 and 2), it failed to elevate the numbers of Osx-positive cells in *Atf4^−/−^* tibiae. Similar results were obtained in calvariae (Fig. 6E). The IHC staining was highly specific since no signal was detected in the non-immune IgG control group (Panel 5). Consistent with IHC results, as shown in Fig. 6I, Western blot analysis using protein extracts showed that PTH dramatically elevated the level of Osx protein in wt tibiae. In contrast, Osx was not detected by Western blot in extracts from *Atf4^−/−^* animals (Fig. 6I). The level of Runx2 protein was slightly up-regulated by PTH in the wt group, but not in *Atf4^−/−^* group. Unlike Osx, the basal level of Runx2 was not reduced by ATF4 deficiency. PTH1R protein, the major receptor for PTH and PTHrP signaling in osteoblasts, was expressed in endosteal osteoblasts of tibiae (Fig. 6F) and periosteal osteoblasts of calvariae and hypertrophic chrodrocytes in the growth plate area (unpublished data). The signal for PTH1R protein was weak in trabecular osteoblasts that actively form new bone (unpublished data). In contrast to results from a previous study showing that PTH1R is down-regulated by PTH in cultured osteoblasts [46], PTH1R was slightly increased by intermittent PTH treatment in vivo as measured by IHC and Western blot analysis. Importantly, ATF4 deficiency did not reduce the level of PTH1R (Fig. 6F and I). In addition, primary calvarial osteoblasts from wt and *Atf4^−/−^* mice displayed an identical cAMP accumulation curve in response to treatment of increasing concentrations of PTH in vitro. Taken together, these results indicate that the impaired anabolic response of skeleton to PTH observed in *Atf4^−/−^* animals cannot be explained by a reduction in the level of PTH1R and/or cAMP production. These results clearly demonstrate that: i) intermittent PTH stimulates the expression of Osx and, to a lesser extent, Runx2, ii) PTH fails to stimulate Osx/Runx2 expression in the absence of ATF4, and iii) ATF4 is also required for basal level Osx expression.

### Identification of a 132-bp ATF4-response element in proximal Osx promoter

**Figure 7:**
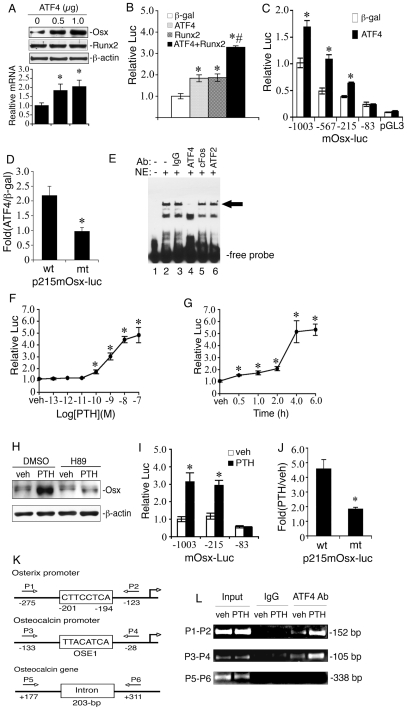
PTH activates Osx gene transcription via an ATF4-responsive element in the proximal Osx promoter. A, MC-4 cells were electroporated with indicated amount of ATF4 expression plasmid followed by Western blot. B, COS-7 cells were transfected with p1060mOsx-luc, pRL-SV40, and indicated expression vectors followed by dual lucferase assays. C, COS-7 cells were transfected with various deletion constructs and pRL-SV40 with and without ATF4 expression plasmid. D, COS-7 cells transfected with p215mOsx-luc or the same plasmid containing a 3-bp substitution mutation in the putative ATF4-binding site and pRL-SV40 with and without ATF4 expression plasmid. E, EMSA, labeled wild-type DNA probe was incubated with 2 µg nuclear extracts from COS-7 cells transfected with pCMV/ATF4 plasmid in the presence of normal control IgG (lane 3), ATF4 antibody (lane 4), cFos antibody (lane 5), and ATF2 antibody (lane 6). Experiments were repeated 3–4 times and qualitatively identical results were obtained. F and G, MC-4 cells transfected with p1003mOsx-luc and pRL-SV40 were treated with indicated concentration of PTH for 6 h (F) or with 10^−7^ M PTH for indicated times (G). H, MC-4 cells were treated with and without 10^−7^ M PTH in the presence and absence of 10 µM of H89 for 6 h. I, MC-4 cells transfected as in Fig. 7C were treated with and without 10^−7^ M PTH for 6 h. J, MC-4 cells transfected as in Fig. D were treated with and without 10^−7^ M PTH for 6 h. K, a schematic illustration of putative ATF4 binding sites in the 5′ flanking regions of the *Osx* and *osteocalcin* gene promoters and *osteocalcin* gene. L, ChIP assay of the *Osx* promoter in MC-4 cells treated with and without 10^−7^ M PTH for 6 h. *P<0.05 (β-gal vs. ATF4, Runx2, and ATF4 plus Runx2, or veh vs. PTH), ^#^P<0.05 (ATF4 plus Runx2 vs. β-gal, ATF4, or Runx2).

Osx is not detected in *Runx2^−/−^* mice [35], indicating that Runx2 functions upstream of this factor and is essential for Osx expression. However, the results described above showed that ATF4 deficiency dramatically reduced the level of Osx protein without decreasing Runx2, suggesting that Runx2 is not sufficient for the maximal expression of Osx and that ATF4 has an important role in Osx expression. To define the mechanism whereby ATF4 regulates Osx, we examined the effect of ATF4 overexpression on Osx expression in MC-4 preosteoblast cells. As shown in Fig. 7A, ATF4 dose-dependently increased levels of Osx protein (top) and mRNA (bottom). We next examined whether ATF4 up-regulates Osx by increasing gene transcription by using a −1003/+68 mouse Osx promoter (Fig. 7 B). Using COS-7 cells, which lack detectable Runx2, ATF4 had comparable activity to Runx2 in terms of its ability to activate promoter activity (approx. 1.8-fold). Together, ATF4 and Runx2 maximally activated the *Osx* promoter (3.2-fold induction). To further define the region of the Osx promoter necessary for ATF4 responsiveness, several constructs containing various deletion mutants of the mouse *Osx* promoter were transiently transfected into COS-7 cells with and without an ATF4 expression plasmid. Results showed that luciferase activity of both control and ATF4-transfected groups decreased with progressively larger 5′ deletions. However, ATF4 stimulation was abrogated when a 132-bp region between bp −215 to −83 was deleted (Fig. 7C). A putative ATF4-binding sequence (CTTCCTCA) at −201/−194 bp was identified in this region by using a TRANSFAC retrieval program. Introduction of a 3-bp substitution mutation to this core sequence (from CTTCCTCA to CTTgtaCA) completely abolished ATF4 activation (Fig. 7D). As shown in Fig. 7E, a DNA oligo probe from the Osx promoter that contains the TTACATCA core sequence bound to a factor(s) in nuclear extracts from COS-7 cells transfected with an ATF4 expression vector. Importantly, this binding (see arrow) was dramatically reduced by the addition of a specific antibody against ATF4 but not by normal control IgG or antibodies against cFos (an AP1 family member) or ATF2.

### PTH stimulation of Osx gene transcription requires an ATF4 response element

To study the mechanism whereby PTH regulates *Osx*, we next evaluated the effect of PTH on mouse −1003/+68 *Osx* promoter activity in MC-4 preosteoblast cells. As illustrated in Fig. 7F, PTH stimulated promoter activity in a dose-dependent manner with a detectable response seen at a PTH concentration of 10^−10^ M (significance at P<0.01). Measurable activation of the *Osx* promoter was observed 0.5 h after PTH addition with maximal induction occurring between 4–6 h (Fig. 7G). PTH-stimulated Osx protein expression was entirely blocked by PKA inhibition (Fig. 7H). The 132-bp ATF4-responsive element identified above was also required for PTH induction of promoter (Fig. 7I). Furthermore, the same 3-bp substitution mutation that abrogates ATF4 activation dramatically reduced PTH-dependent activation (Fig. 7J), indicating that this element is critical for the actions of PTH on this promoter.

### ATF4 is recruited to the endogenous Osx promoter in a PTH-dependent manner

To determine whether ATF4 is associated with the endogenous *Osx* promoter in vivo, we performed chromatin immunoprecipitation (ChIP) assays using MC-4 cells with and without PTH treatment. As shown in Fig. 7K and L, ATF4 specifically interacted with a chromatin fragment of the proximal *Osx* promoter that contains the ATF4-binding site identified above. This interaction was not detected in primary calvarial osteoblasts from *Atf4^−/−^* mice (data not shown). Furthermore, this interaction was dramatically stimulated by PTH treatment. Supporting our previous demonstration that the OSE1 site in the *mOG2* promoter mediates PTH induction of the gene [31], [34], ATF4 also bound to an ATF4-binding site (OSE1)-containing chromatin fragment of the proximal *mOG2* promoter in a PTH-dependent manner (primers P3/P4). In contrast, ATF4 antibody failed to immunoprecipitate a 3′ chromatin fragment in the transcribed region of the *mOG2* gene that contains no ATF4-binding sites (primers P5/P6).

## Discussion

Our goals in this study were: 1) to determine whether the bone-related transcription factor ATF4 plays a role in the anabolic effects of PTH in bone, and 2) if so, to define the relevant mechanisms. Our results clearly show that PTH-stimulated increases in osteoblast proliferation, volume of long bones, vertebrae, and calvariae as well as decreases in apoptosis are all dramatically reduced or completely abolished in *Atf4*-deficient mice. Equally importantly, PTH-induced bone in *Atf4* ^−/−^ mice cannot mature due to a severe defect in osteoblast differentiation as manifested by a defect in Osx expression. Therefore, this study establishes a critical role for ATF4 in the anabolic actions of PTH in bone.

In agreement with results from previous studies [14], [47], we find that intermittent PTH dramatically increases trabecular and cortical bone volume. This PTH response is achieved at least in part by stimulating the proliferation of osteoblasts and preosteoblasts and/or by inhibiting apoptotic death of osteoblasts and osteocytes in vivo [2], [6], [45], [47]. Effects of PTH on osteoblast proliferation and survival can be reproduced in cultured osteoblasts [15], [16], [48]. Importantly, this study demonstrates that ATF4 plays a pivotal role in PTH stimulation of cell proliferation in osteoblasts and preosteoblasts and attenuation of apoptosis in osteoblasts and osteocytes in vivo. PTH can increase osteoblast proliferation at least in part through ATF4-mediated expression of cyclin D1 [33] because both factors are up-regulated by PTH in osteoblasts [15], [34]. The mechanism whereby ATF4 blocks apoptosis in osteoblasts remains unknown. ATF4 deficiency also increases apoptosis in lens fiber cells in a p53-dependent manner. The embryonic lens in double homozygous *p53/Atf4^−/−^* mice does not undergo apoptosis, which suggests possible involvement of p53 in this process [49], [50].

Accumulating evidence supports the concept that, in addition to increasing osteoblast cell number, intermittent PTH also stimulates osteoblast differentiation [4], [51]–[53]. In agreement with these results, the present study clearly demonstrates that: i) PTH dramatically increases the in vivo expression of osteoblast differentiation marker genes, including *Ocn*, *Bsp*, *Alp*, *Opn*, and *Col1(I)*; and ii) PTH strikingly elevates the numbers of Osx-positive osteoblasts (i.e., mature or differentiating osteoblasts) and level of Osx protein as demonstrated by both IHC and Western blot analysis. Because PTH is able to stimulate the expression of many osteoblast differentiation marker genes in cultured osteoblast-like cells [12], [30], [31], [54]–[57], it is likely that intermittent PTH also activates these genes in vivo via a similar molecular mechanism. Although part of the increased osteoblast activity in PTH-treated animals is likely explained by a PTH-dependent increase in osteoblast proliferation and/or reduction of apoptosis, our studies suggest that PTH also increases osteoblast differentiation by rapidly up-regulating Osx.

Importantly, experiments from the current study show that effects of PTH on osteoblast differentiation are mediated by ATF4. Although PTH increased osteoblast proliferation in *Atf4^−/−^* animals as evidenced by significant increases in bone volume and nuclear BrdU labeling (Figs. 1–3), expression of osteoblast marker genes and numbers of Osx-positive osteoblasts were either dramatically reduced or completely abolished in *Atf4^−/−^* animals. Furthermore, since the basal levels of osteoblast differentiation markers are either slightly reduced or not changed at all in *Atf4^−/−^* animals, the impaired differentiation response to PTH cannot be explained by a nonspecific blockage in osteoblast differentiation associated with ATF4 deficiency. Instead, ATF4 appears to have a unique role in the PTH-dependent component of osteoblast differentiation.

Further support for the concept that PTH actions are mediated by ATF4 comes from mechanistic studies. Specifically, we showed that PTH is a potent inducer of Osx expression in vivo and this response is completely abolished by ATF4 deficiency. Furthermore, PTH directly activated *Osx* gene transcription in cultured osteoblast-like cells, a response that required an ATF4 response element located between −201 and −194 bp in the proximal mouse *Osx* promoter. Introduction of a 3-bp substitution mutation into this ATF4-binding site essentially eliminated the PTH response. ChIP assays demonstrated that ATF4 binds to an endogenous chromatin fragment near the putative ATF4-binding site in the proximal *Osx* promoter in MC-4 cells. Of particular significance, ATF4 binding to the *Osx* promoter is dramatically enhanced by PTH. Collectively, these studies establish a unique role for ATF4 in PTH-mediated induction of Osx and osteoblast differentiation.

Runx2 is absolutely required for Osx expression, osteoblast differentiation, and bone formation [35], [58]. Nevertheless, ATF4 deficiency dramatically reduced the level of Osx protein without altering Runx2, suggesting that Runx2 is not sufficient for maximal Osx expression. ATF4 stimulated Osx gene transcription in COS-7 cells that lack Runx2 protein to an extent similar to that seen with Runx2. In addition, an ATF4 response element was identified in the proximal region of the mouse Osx promoter. These results demonstrate that both ATF4 and Runx2 are essential for the maximal expression of Osx and are reminiscent of our previous study showing cooperative interactions between these two factors in regulating the *Ocn* gene [59].

It should be noted that ATF4 deficiency did not completely block the anabolic actions of PTH since this hormone increased bone volume in both growing and adult OVX bones from *Atf4* ^−/−^ mice (Figs. 1–3). This suggests that other factors must be involved for the PTH response. c-Fos [3], cAMP response element binding protein (CREB) [16], a major downstream target for PTH/cAMP and calcium signals, cAMP-response element modulator (CREM)[60], and Runx2 [16], [23], a master regulator of osteoblast differentiation and bone formation, have all been shown to mediate components of the PTH anabolic response. Interestingly, these factors are either structurally related to ATF4 (c-Fos and CREM) or can interact with this factor (Runx2 and c-Fos) [59], [61]–[65]. Most recently, low-density lipoprotein-related protein 6 (LRP6)[66], a major component of the Wnt signaling pathway, has been implicated in the anabolic actions of PTH in bone. It would be interesting to determine whether ATF4 mediates the PTH anabolic response via interactions with these factors or signaling pathways.

PTH signaling may regulate ATF4 via several mechanisms. First, PTH up-regulates *Atf4* gene expression in cultured osteoblasts as demonstrated by our recent study [34] as well as in vivo (Fig. 5). Second, PTH post-translationally activates ATF4 via PKA [34], a major route for PTH signaling in osteoblasts. PKA phosphorylation of ATF4 at its Ser254 residue mediates β-adrenergic induction of *Rankl* mRNA expression in osteoblasts [67]. ATF4 can also be directly phosphorylated and activated by RSK2 [26], a growth factor-regulated serine-threonine protein kinase activated by the Ras-Mitogen-Activated Protein Kinase (MAPK) pathway. This phosphorylation is critical for ATF4 activity as well as bone formation [26]. Because PTH signaling activates Erk/MAPK [48], an immediate upstream activator of RSK2, ATF4 can be activated via the PTH-MAPK-RSK2 signaling pathway. Lastly, PTH promotes ATF4-Runx2 interactions which are critical for osteoblast function and bone formation [59], [61], [63]. This notion is supported by the fact that PTH up-regulates both factors in osteoblasts [23], [34].

**Figure 8:**
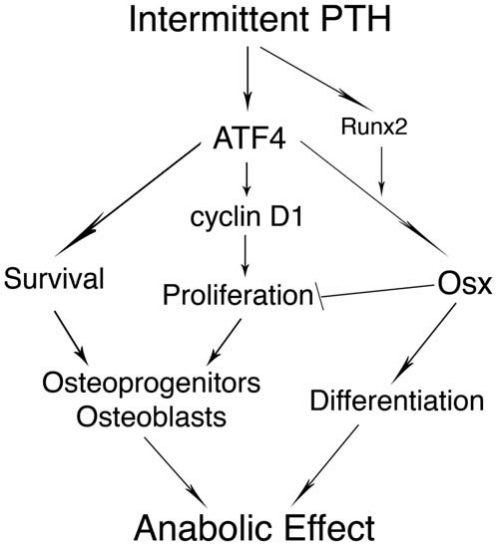
Proposed model for ATF4 mediation of PTH stimulation of bone formation. Binding of PTH to PTH1R activates PKA and leads to up-regulation of ATF4. ATF4 subsequently increases proliferation and survival of osteoblasts. At the same time, ATF4 together with Runx2 maximally activates Osx expression and increases osteoblast differentiation. These increases in osteoblast number and differentiation lead to massive bone formation. Osx also negatively regulates osteoblast proliferation, thus preventing excess bone formation.

Based on findings from this and other studies, we proposed a working model for ATF4 to mediate PTH stimulation of osteoblast function and bone formation (Fig. 8). Binding of PTH to its receptor, PTH1R, activates PKA and probably other intracellular signaling pathways, leading to up-regulation/activation of ATF4. ATF4 subsequently increases proliferation of osteoblasts and/or preosteoblasts via modulation of cyclin D1 protein, and attenuates apoptotic death in osteoblasts and osteocytes, resulting in a significant increase in the numbers of osteoblasts and/or osteocytes. At the same time, ATF4 together with Runx2 maximally activates Osx expression and increases osteoblast differentiation. The resulting increases in osteoblast number and differentiation lead to bone formation. Osx also negatively regulates osteoblast proliferation, thus preventing excess bone formation [68].

Since ATF4 is known to regulate the expression of RANKL in osteoblasts and thereby osteoclast differentiation [67], [69], it will be interesting to determine if ATF4 is also required for the catabolic actions of PTH in bone.

In summary, this study establishes a critical role for ATF4 in the anabolic actions of PTH in bone. ATF4 is necessary for PTH to increase both osteoblast numbers and differentiation. Therefore, ATF4 may provide a potential new therapeutic target for improving bone mass and for treating metabolic bone diseases such as osteoporosis.

## Materials and Methods

### Reagents

Tissue culture media and fetal bovine serum were obtained from HyClone (Logan, UT). H89, DMSO, PTH1R antibody, mouse monoclonal antibody against β-actin were purchased from Sigma (St Louis, MO). Other reagents were obtained from the following sources: antibodies against ATF4 (for Western blot), Runx2, normal control IgGs and horseradish peroxidase-conjugated mouse or goat IgG from Santa Cruz (Santa Cruz, CA), and Osterix antibody from Abcam Inc. (Cambridge, MA). ATF4 antibody used for EMSA was raised against epitope QETNKEPPQTVNPIGHLPESLIK (St Louis, MO). All other chemicals were of analytical grade.

### Atf4-deficient mice

Breeding pairs of *Atf4* heterozygous mice were described previously [34] and used to generate *Atf4* wild-type (wt) (*Atf4* ^+/+^), heterozygous (*Atf4* ^+/−^) and homozygous mutant (*Atf4* ^−/−^) mice for this study. All research protocols were approved by the Institutional Animal Care and Use Committee of the VA Pittsburgh Healthcare System, where this study was conducted.

### In vivo PTH administration

For the “growing mouse model”, five-day-old mice were given daily subcutaneous injections of vehicle (saline) or hPTH(1–34) (60 ng/g body weight, Bachem, Torrance, CA) for 28 d. For the “adult OVX mouse model”, four-month-old female mice were anesthetized and ovariectomized as follows: A 1-cm midline incision was made through the skin. The “white line” will be visualized on the peritoneum and a second incision was made along the white line through the peritoneum. Using long, straight forceps, the left ovary was isolated and the connective tissue between the ovary and kidney was dissected away. Straight forceps with flat ends was used to pinch the uterine horn while another set of straight forceps was used to tear the ovary away from the uterine horn. The same was done on the right ovary. 6-0 PDS (Polydioxanone Sutures, 6/0) was used to tie around the uterine horn to provide hemostasis if necessary. 6-0 PDS was used to close the innermost layer. Sterile surgical staples were used to close the incision. Two months later mice were given daily subcutaneous injections of vehicle or hPTH(1-34) (100 ng/g body weight) for 28 d. Mice were euthanized 24 h after last PTH injection. The effects of these PTH dosing regimens on bone were determined by both biochemical and histomorphometric criteria.

### Gross evaluation and serum biochemistry

Body weight was recorded every another day. The length of the femurs was measured using an electronic digital caliper. Faxitron X-ray analysis of femurs was conducted at 27 kv and 7.5 seconds (Faxitron X-Ray Corp., Wheeling, IL). Femurs were ashed at 800°C for 4 h and weighed. Serum calcium and Pi concentrations were determined using kits from Pointe Scientific, Inc (Canton, MI) following the manufacture's instructions (Sigma Diagnostics).

### Bone morphometric analyses by micro-computerized tomography (μC*T*)

Upon termination of PTH or vehicle treatment, mice were sacrificed and femurs were isolated. Fixed non-demineralized femurs were used for μCT analysis at the Center for Bone Biology using a VIVACT40 (SCANCO Medical AG) following the standards of techniques and terminology recommended by American Society for Bone and Mineral Research [70]. For trabecular bone parameters, transverse CT slices were obtained in the region of interest in the axial direction from the trabecular bone 0.1 mm below the growth plate (bottom of the primary spongiosa) to the mid-femur. Contours were defined and drawn close to the cortical bone. The trabecular bone was then removed and analyzed separately. 3D analysis was then performed on trabecular bones slices. A 3-mm section was used to obtain mid-femoral cortical bone thickness. The analysis of the specimens involves the following bone measurements: bone volume fraction (BV/TV, %), trabecular number (Tb. N), trabecular thickness (Tb. Th), trabecular spacing (Tb. Sp), and cortical thickness (Cort.Th).

### Histological evaluation

Tibiae, lumbar vertebrae (L5), and calvariae were fixed in PBS buffered 10% formalin at 4°C for 24 h, decalcified in 10% EDTA (pH 7.4) for 10–14 d, and embedded in paraffin. Longitudinal sections of tibiae and vertebrae were cut at 4 µm and stained with hematoxylin and eosin (H&E). Trabecular area of tibial sections was measured in the proximal metaphysis beginning immediately below the chondro-osseous junction to the mid-tibia. Calvariae were bisected perpendicular to the sagittal suture through the central portion of the parietal bones, parallel to lamboidal and coronal sutures, and embedded in paraffin to obtain sections of a standard area according to the method described by Zhao et al. [71]. Trabecular area versus total bone area was measured using an Image Pro Plus 6.2 software (Media Cybernetics, Inc, Bethesda, MD). The calvarial width was the average value from 20 random measurements of each calvaria (at least 6 samples per group) using a SPOT Advanced imaging software (provided with the purchase of the Olympus BX41 microscope).

### Measurement of mineral apposition rate (MAR)

Mice were injected with calcein subcutaneously (20 mg/kg) at 6 and 2 d before sacrifice. Undecalcified tibia were fixed in 70% ethanol, embedded in methylmethacrylate and sectioned at 10 µm. Calcein labeling was visualized using a Nikon E800 fluorescence microscope. The metaphyseal trabecular bone projected into the marrow space was evaluated and the distance between the all double-labeled areas was measured at a magnification of 200x. MAR was calculated as mean distance between the double labels divided by the number of the days between the calcein injections. Histomorphometric analysis was performed using BioQuant image analysis software (R&M Bio Metrics, Nashville, TN, USA).

### In vivo proliferation assay

Mice were injected intraperitoneally with 100 µg bromodeoxyuridine (BrdU)/12 µg fluorodeoxyuridine (FdU) per gram of body weight 12 h before sacrifice. After sacrifice, sections of tibiae and calvariae were obtained. To identify actively proliferating cells, nuclei that have incorporated BrdU were detected using a Zymed BrdU immunostaining kit according to the manufacturer's instruction (Invitrogen, Carlsbad, CA). BrdU-positive cells (brown) on the calvarial periosteal surface or in the osteoid of tibiae were counted and normalized to the total numbers in the same area [33]. BrdU-positive hematopoietic cells in marrow were not counted.

### In situ apoptosis detection

This assay is based on the classical TUNEL assay to examine apoptosis by detecting DNA fragmentation. 4-µm sections of tibiae were prepared and stained using the ApopTag Peroxidase *In Situ* Apoptosis Detection Kit according to the manufacturer's instruction (Millipore, Billerica, MA). Apoptotic osteoblasts and osteocytes in tibiae were counted and normalized to the total cells from the same area.

### Immunohistochemistry (IHC)

Tibiae and calvariae were fixed, decalcified, and embedded in paraffin. Sections of tibiae and calvariae were stained with antibodies against Osx (Abcam Inc, Cambridge, MA) and PTH1R (Sigma, St. Louis, MO) using the EnVision^+^System-HRP (DAB) kit (Dako North America, Inc, Carpinteria, CA) according to the manufacturer's instructions. Briefly, slides were baked at 55°C for 45 min, deparaffinized in three washes of xylene, and rehydrated in a decreasing ethanol gradient. Antigen retrieval was performed using 0.1% trypsin for 10 min at 37°C in a humidified chamber. Endogenous peroxidases were deactivated with 3% H_2_O_2_ in 1x PBS for 10 min, and sections were blocked in blocking solution for 30 min at room temperature. Sections were incubated with primary antibody (1∶200 dilution for both osterix and PTH1R) in blocking solution for 2 hours at 4°C. Sections were washed in PBS three times and incubated with a donkey-anti-rabbit IgG-HRP secondary antibody solution for 30 min at room temperature. After washing with PBS three times, HRP activity was detected using a DAB substrate solution for 5 min at room temperature. Sections were counter-stained with a Mayer's hematoxylin solution.

### Measurement of plasma levels of IGF-1 and FGF-2 by ELISA

Blood plasma samples were prepared from whole-blood samples from mice of each group and plasma levels of IGF-1 and FGF-2 were measured by using ELISA kits (human FGF basic Quantikine ELISA Kit, cat#: DFB50, and mouse IGF-I Quantikine ELISA Kit, cat#: MG100, both from R&D Systems Inc, Minnepolis, MN 55413) according to the manufacturer's instructions.

### cAMP assay

Primary osteoblasts from calvariae of 3-d-old wt or *Atf4^−/−^* mice were isolated as described previously [72]. Cells were seeded at a desity of 5×10^4^/well on 96-well plate and treated with vehicle or increasing concentrations of human recombinant PTH(1-34) for 5 min. Cells were then lysed with lysis reagent 1B and cell lysates used for cAMP assay using a cAMP Biotrak Enzymeimmunoassay (EIA) kit (cat #: RPN225, GE Healthcare Biosciences Corp, Piscataway, NJ) according to the manufacturer's instructions and the protein concentrations were measured using a BCA protein assay kit (Pierce). cAMP was normalized to total protein.

### Quantitative real-time RT/PCR and Western blot analysis

RNA isolation, reverse transcription (RT), regular PCR, and quantitative real-time PCR analysis were performed as previously described [34]. The DNA sequences of mouse primers used for real-time PCR were summarized in Table 1. Western blot analysis was performed as previously described [33]. RNAs or protein extracts from at least six specimens in each group were used.

**Table 1:** real-time PCR primers.

Gene name	5′ primer	3′ primer
*Alp*	TCCCACGTTTTCACATTCGG	CCCGTTACCATATAGGATGGCC
*Atf4*	GAGCTTCCTGAACAGCGAAGTG	TGGCCACCTCCAGATAGTCATC
*Bsp*	AAGAGGAAGAAAATGAGAACGA	GCTTCTTCTCCGTTGTCTCC
*cFos*	AATGGTGAAGACCGTGTCAGGA	CCCTTCGGATTCTCCGTTTCT
*cJun*	GCCAACATGCTCAGGGAACAGGTG	GCCCCTCAGCCCTGACAGTCTG
*Col I(1)*	AGATTGAGAACATCCGCAGCC	TCCAGTACTCTCCGCTCTTCCA
*Gapdh*	CAGTGCCAGCCTCGTCCCGTAGA	CTGCAAATGGCAGCCCTGGTGAC
*Ocn*	TAGTGAACAGACTCCGGCGCTA	TGTAGGCGGTCTTCAAGCCAT
*Opn*	CCAATGAAAGCCATGACCACA	CGTCAGATTCATCCGAGTCCAC
*Osx*	AGAGGTTCACTCGCTCTGACGA	TTGCTCAAGTGGTCGCTTCTG
*Pth1r*	GATGCGGACGATGTCTTTACC	GGCGGTCAAATACCTCC

### DNA constructs and site-directed mutagenesis, transfection

pCMV/β-gal, pCMV/ATF4, and pCMV/Runx2 were previously described [62]. mOsx-luc containing different mouse *Osx* promoter elements (−1003/+68, −567/+68, −215/+68, and −83/+68) driving a firefly luciferase reporter gene were constructed in the project laboratory by PCR subcloning promoter fragments using mouse tail DNA as a template into pGL3-luc vector (Promega, Madison, WI). Mutant p215mOsx-luc which contains a 3-bp substitution mutation in a putative ATF4-binding at positions −198, −197 and −196 (from CTTCCTCA to CTTgtaCA) was generated from the wild-type p215mOsx-luc by PCR amplification using a QuickChange^TM^ XL Site-Directed Mutagenesis Kit (Stratagene, La Jolla, CA) using the following primers: 5′-GGT ACC CCT CCC TCT CTC GCC TTg taC ATT GGA TCC GGA GTC TTC TCC GC-3′ (forward); 5′-GCG GAG AAG ACT CCG GAT CCA ATG tac AAG GCG AGA GAG GGA GGC GGT ACC-3′ (reverse). Sequence accuracy was confirmed by automatic DNA sequencing. For all transfection experiments, the amount of plasmid DNAs (reporter plasmid, 0.25 µg; normalization plasmid pRL-SV4, 10 ng; and expression plasmid, 1.0 µg) was balanced as necessary with β-galactosidase expression plasmid such that the total DNA was constant in each group. Experiments were performed in triplicates and repeated 3–4 times.

### Nuclear extracts preparation and electrophoretic mobility shift assay (EMSA)

Nuclear extracts were prepared from COS-7 cells transfected with pCMV/ATF4 plasmids as previously described [73]. The DNA sequences of the oligonucleotides used for EMSA were as follows: GAT CCC TGC CTC CCT CTC TCG CCT TCC TCA TTG GAT CCG GAG TCT TCG. DNA oligonucleotide was labeled using a Biotin 3′ end DNA Labeling Kit (cat #: 89818, Pierce Biotechnology Inc., Rockford, IL). Two µg of nuclear extracts and 20 fmol biotin-labeled DNA probe were incubated in 1x binding buffer for 30 min at room temperature. For supershift assay, 1 µg of IgG or indicated antibodies were first incubated with nuclear extracts prior to addition of DNA probe. Protein–DNA complexes were separated on 4% polyacrylamide gels in 1x TBE buffer, and transferred onto Biodyne B Nylon Membrane (cat #: 77016, Pierce). The membrane was blocked in 1x blocking buffer, washed five times with 1x wash buffer, and visualized by a Chemiluminescent Nucleic Acid detection Module (cat #: 89880, Pierce, Rockford, IL).

### Chromatin immunoprecipitation (ChIP)

ChIP assays were performed using ATF4 antibody or control IgG as described previously [62]. PCR primer pairs (Table 2) were generated to detect DNA segments located near a putative ATF4-binding site (CTTCCTCAT) at −201/−193 (primers P1 and P2) determined by the TRANSFAC retrieval program in the 5′ flanking region of the *Osx* promoter, a previously identified ATF4-binding site (OSE1) in *osteocalcin* gene 2 (mOG2) promoter (primers 3 and 4), and a mOG2 gene region (+177/+311) that contains no ATF4-binding sites (primers P5 and P6) [62]. PCR products were run on 3% agarose gel and stained with ethidium bromide. Purified input chromatin was used to perform parallel PCRs with the respective primer pairs.

**Table 2:** PCR primers used in ChIP assay.

Oligo name	Sequence
P1	CCCTCCCAGATCCCTTCTTT
P2	GGCTGCTCTCTGTCTGTAGGG
P3	CACAGCATCCTTTGGGTTTGAC
P4	TATCGGCTACTCTGTGCTCTCTGA
P5	TAGTGAACAGACTCCGGCGCTA
P6	TGTAGGCGGTCTTCA AGCCAT

### Statistical analysis

Data was analyzed with a GraphPad Prism software (4.0). A one-way ANOVA analysis was used followed by the Tukey test. Students' *t* test was used to test for differences between two groups of data as needed. Data of Figs. 1, 2, 4, and 5 and S1-2 were from growing mice and Figs. 3 and S3 from 7-month-old OVX mice. For H&E, IHC, BrdU, and TUNEL studies, two to three sections per specimen and at least six specimens in each group were used. Results were expressed as means ± standard deviation (SD). Differences with a P<0.05 was considered as statistically significant.

## Supporting Information

Figure S1 Effects of PTH on animal growth, length and ash weight of femurs, and serum Pi and calcium concentrations and alkaline activity in wt and Atf4−/− mice. A, growth curve, B, length of femur, C, dry ash weight of femur, D, serum Pi, E, serum calcium. *P<0.05 (veh vs. PTH), † P<0.05 (wt-veh vs. Atf4-/--veh).
(1.78 MB TIF)

Figure S2 Faxitron X-ray analysis of femurs from vehicle and PTH-treated growing wt, Atf4+/−, and Atf4-/− mice. Faxitron X-ray analysis was conducted at 27 kv and 7.5 seconds. Representative microradiographic images of femurs are shown.
(1.45 MB TIF)

Figure S3 Effects of OVX surgery on bone parameters in wt and Atf4−/− mice. Four-month-old female mice were first ovariectomized. After two months, femurs were isolated for μCT analysis. *P<0.05 (sham vs. OVX), † P<0.05 (sham(+/+) vs. sham (−/−)).
(0.16 MB TIF)
