# Supplementary figures and images for: Critical Role of Activating Transcription Factor 4 in the Anabolic Actions of Parathyroid Hormone in Bone
Source: PLoS One. 2009 Oct 23;4(10):e7583. doi: 10.1371/journal.pone.0007583 (PMC2762317; doi:10.1371/journal.pone.0007583)

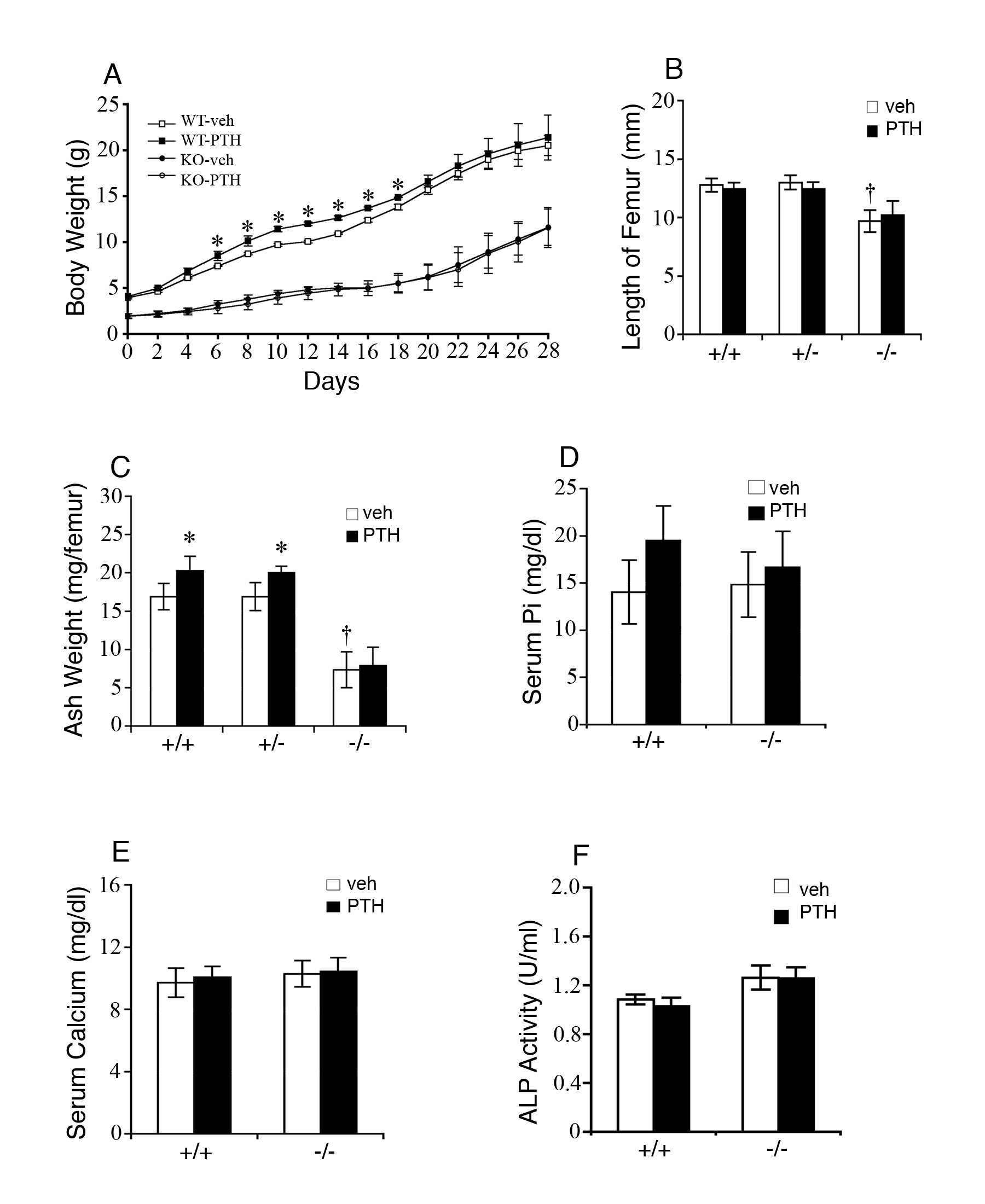

Supplement: Figure S1 — Effects of PTH on animal growth, length and ash weight of femurs, and serum Pi and calcium concentrations and alkaline activity in wt and Atf4−/− mice. A, growth curve, B, length of femur, C, dry ash weight of femur, D, serum Pi, E, serum calcium. *P<0.05 (veh vs. PTH), † P<0.05 (wt-veh vs. Atf4-/--veh). (1.78 MB TIF) [file pone.0007583.s001.tif]

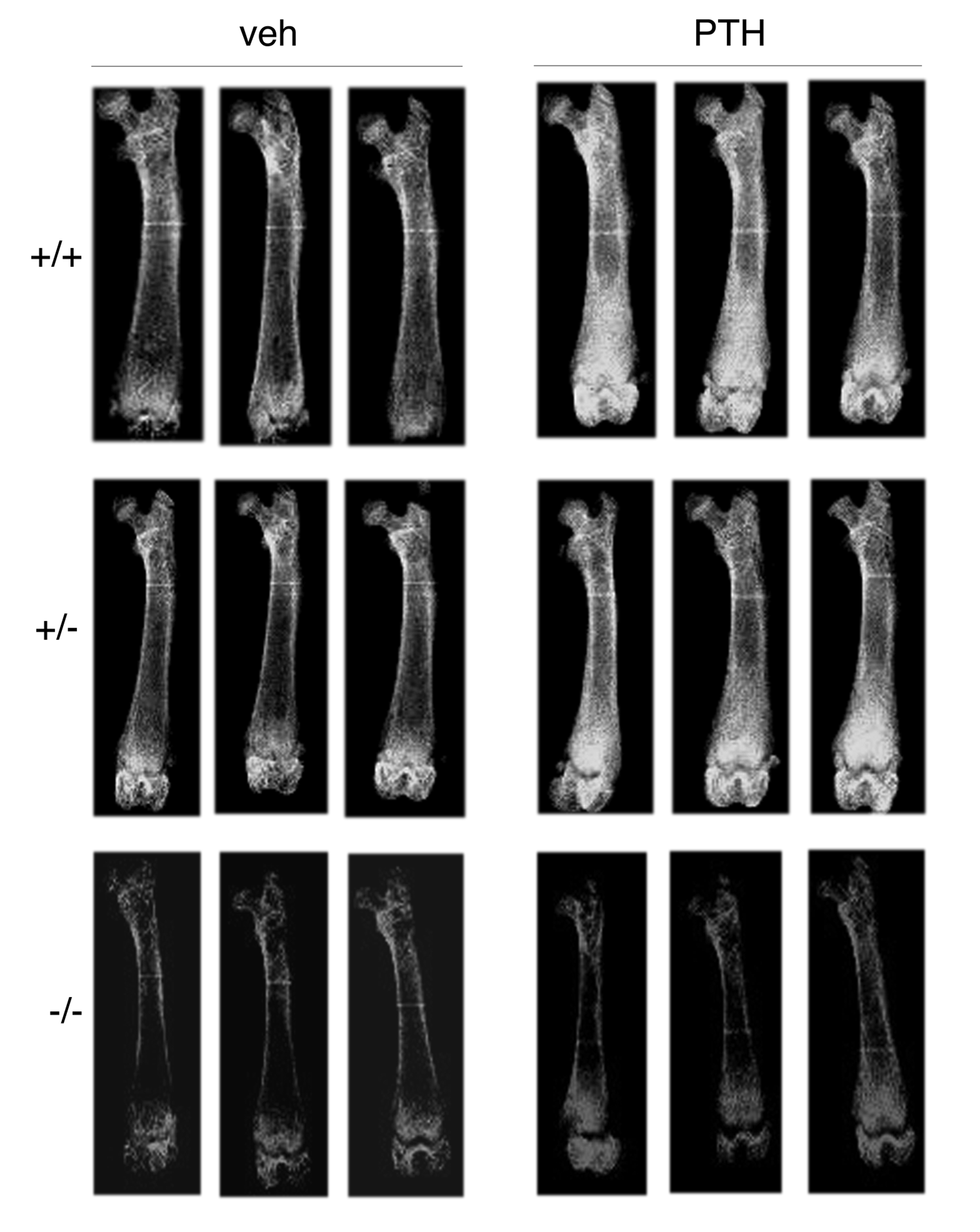

Supplement: Figure S2 — Faxitron X-ray analysis of femurs from vehicle and PTH-treated growing wt, Atf4+/−, and Atf4-/− mice. Faxitron X-ray analysis was conducted at 27 kv and 7.5 seconds. Representative microradiographic images of femurs are shown. (1.45 MB TIF) [file pone.0007583.s002.tif]

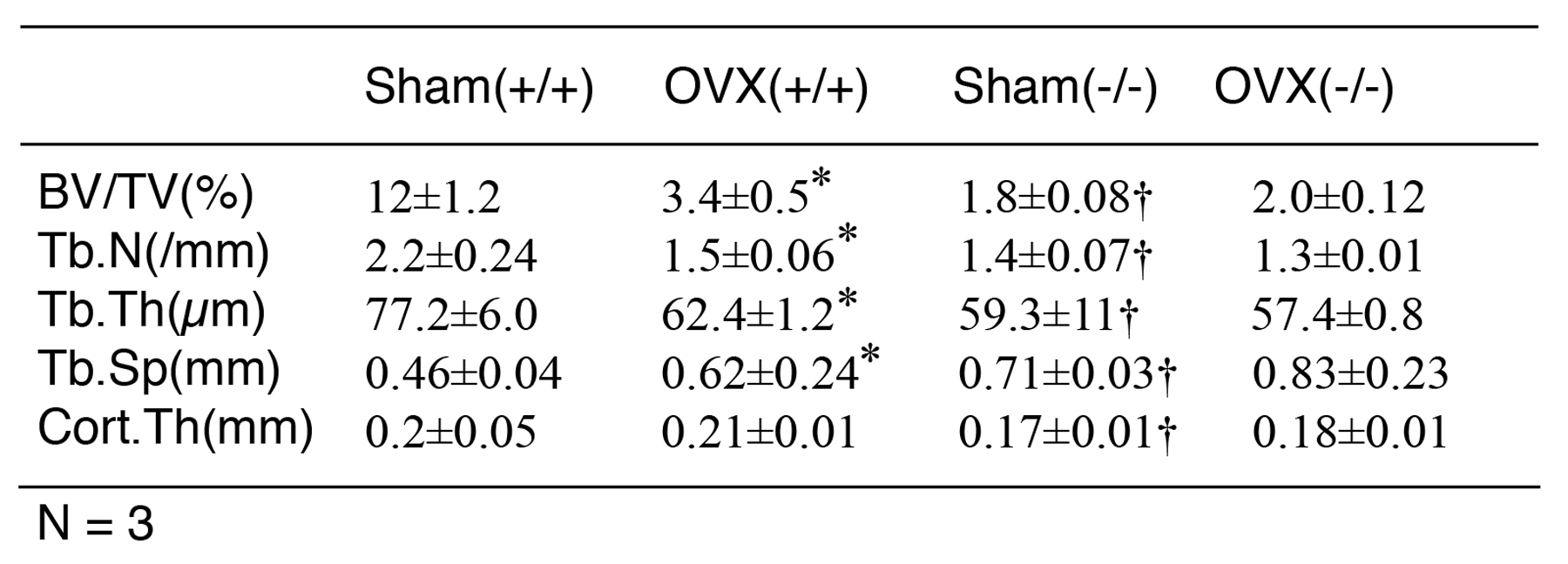

Supplement: Figure S3 — Effects of OVX surgery on bone parameters in wt and Atf4−/− mice. Four-month-old female mice were first ovariectomized. After two months, femurs were isolated for μCT analysis. *P<0.05 (sham vs. OVX), † P<0.05 (sham(+/+) vs. sham (−/−)). (0.16 MB TIF) [file pone.0007583.s003.tif]
